# An optimized high-quality DNA isolation protocol for *spodoptera frugiperda* J. E. smith (Lepidoptera: Noctuidae)

**DOI:** 10.1016/j.mex.2021.101255

**Published:** 2021-02-03

**Authors:** Diana Victoria Marín, Diana Katherine Castillo, Luis Augusto Becerra López-Lavalle, Jairo Rodríguez Chalarca, Cristo Rafael Pérez

**Affiliations:** a Universidad Nacional de Colombia, Sede Palmira, Palmira, Valle del Cauca, Colombia; b The Alliance of Bioversity International and the International Center for Tropical Agriculture (CIAT), Recta Cali-Palmira km 17, Valle del Cauca, Colombia; c Fedearroz-Fondo Nacional del Arroz, Monteria, Córdoba, Colombia

**Keywords:** Fall armyworm, DNA, COI, Barcoding, DNA isolation, CTAB method

## Abstract

An optimized high-quality DNA isolation protocol was developed using body segment tissue from the Fall Armyworm (Spodoptera frugiperda), that will allow documenting genetic variability based on biotypes, facilitating studies on the appearance, distribution and population dynamics of the fall armyworm at the molecular level. The resulting protocol is an easy-to-use, timesaving method that can rapidly achieve high quality, high-yielding total genomic DNA, using chemicals and everyday consumables available in a molecular laboratory. This new method of DNA extraction avoids the contamination of polysaccharides, salts, phenols, proteins and other cellular by-products that can interfere with subsequent reactions. DNA purity estimates reveal A260: A280 ratios greater than 1.9, which were evidenced by quality test on agarose gel, observing complete integrity and high purity of the resulting samples, and yielded 30–99 µg/g of total DNA. Therefore, the quality of the DNA produced from this extraction is suitable for subsequent molecular applications: (i) next generation whole genome sequencing, (ii) conventional polymerase chain reaction for genotyping, (iii) barcodes and (iv) gene cloning. In addition, to become an anticipating diagnostic tool for invasive lepidopteran larval stages:

• The resulting protocol is an easy-to-use time-saving method.
• This new extraction method prevents contamination from polysaccharides, salts, phenols, proteins, and other cellular sub-products.
• DNA purity estimations reveal A260:A280 ratios above 1.9.

## Graphical abstract

**Figure:**
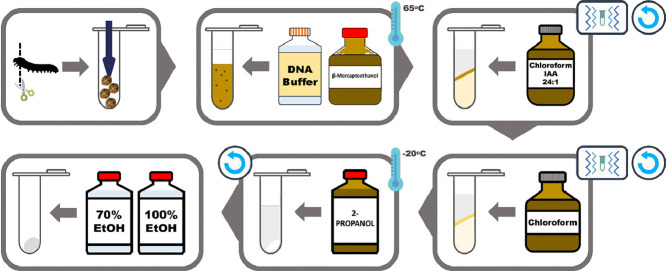

Specifications table

**Table:** 

Subject Area:	Agricultural and Biological Sciences
More specific subject area:	*Molecular entomology*
Method name:	*DNA isolation standardization for fall armyworm*
Name and reference of original method:	*Doyle, J. (1991). DNA protocols for plants. In Molecular techniques in taxonomy (pp. 283–293). Springer, Berlin, Heidelberg.*
Resource availability:	***Reagents:*** *Ribonuclease A from bovine pancreas (Merck; Cat. No. R4875)* *UltraPure™ 1* *M Tris–HCl (*pH *8.0; Thermo Fisher Scientific; Cat. No. 15,568,025)* *Sodium chloride solution 5* *M (Merck; Cat. No. S5150)* *UltraPure™ 0.5* *M ethylenediamine tetraacetic acid (EDTA;* pH *8.0; Promega; Cat. No. V4233)* *Hexadecyltrimethylammonium bromide (CTAB; Merck; Cat. No. H5882)* *2-mercaptoethanol (Sigma-Aldrich; Cat. No. M6250)* *UltraPure™ DNase/RNase-free distilled water (Thermo Fisher Scientific; Cat. No. 10,977–015)* *Chloroform-isoamyl-alcohol mixture 24:1 (Merck; Cat. No. C0549)* *Absolute ethanol for analysis (Merck; Cat. No. 1.00983)* *Chloroform for analysis (Merck; Cat. No. 366,919)* *2-propanol (Merck; Cat. No. 109,634)* ***Materials****:* *Nanodrop^Ⓡ^ ND-1000 spectrophotometer (Thermo Fisher Scientific)* *ELIMINase (Decon Laboratories Inc.; Cat. No. 1101)* *VWR^Ⓡ^ Mini Shaker* *Eppendorf Centrifuge 5427 R, Germany*

## Method details

 

## Introduction

The fall armyworm, *Spodoptera frugiperda* (Lepidoptera: Noctuidae), is the most important polyphagous pest from an economic point of view in South America, and it is associated with more than 27 plant families [1]. The distribution of *S. frugiperda* has changed dramatically from its first report in Africa (Senegal) in January 2016 on maize [2]. To date, *S. frugiperda* has been reported in more than 30 countries in Africa [3]. It was reported during May and June 2018 in Asia [4]. In China alone, in January 2019, *S. frugiperda* was reported in Yunnan Province and by May it had been reported in more than 13 provinces [5]. For S*. frugiperda*, two biotypes (rice and maize) are reported [6,7], which exhibit identical morphological characteristics although they differ in genetic, physiological, and reproductive isolation characteristics. In these two biotypes, cytochrome oxidase I and II and ITS1 genes have been sequenced in different populations of the United States, Mexico, Colombia, Brazil, Argentina, and Paraguay [6,[8], [9], [10].

Therefore, the early detection and monitoring of economically important pests is critical for preventing their dissemination and establishment in valuable commodity crops [11], [12], [13], [14]. To accomplish this, tools for rapid and accurate species identification are needed. DNA-based species identification using short DNA-barcode sequences (500–700 bp) such as mitochondrial cytochrome *c* oxidase subunit I–*CO*I in animals has proven effective for accurate species identification [15,16]. Furthermore, DNA barcoding has shown its potential as a tool for rapid and accurate detection of economically important insects [17], [18], [19].

Similarly, the quality of the DNA sequencing is an important criterion to achieve an accurate identification of the targeted biological entity; hence, access to a high-quality DNA sample is imperative. Thus, a reliable, easy-to-use, fast, and inexpensive DNA extraction method is critical as a first step in generating accurate high-quality DNA barcodes. Otherwise, this can represent a limiting factor in the implementation of barcoding studies. Hence, it is crucial to have access to a time-saving/cost-effective high-quality DNA isolation method that effectively removes contaminants such as polysaccharides, salts, phenols, proteins, and other cellular sub-products [20].

For most Hemiptera species, high-yield/-quality DNA isolation is severely affected by a variety of inhibitory compounds found in the DNA extraction matrix, mainly coming from the processed biological sample (e.g., polysaccharides or phenols) or the chemical used for making the DNA accessible (e.g., CTAB) [21], [22], [23]. However, few studies deal with a comparison of extraction methods intended to find the most appropriate method to use in each species or family [21,^24^,25]. These insects contain plant phenolics and tannins in their digestive tracts, especially when the insects are not adults, and they are not easily dissected for DNA isolation. Phenolics and other secondary compounds cause direct damage to DNA or inhibit enzymatic activity in downstream molecular analysis, particularly restriction endonucleases or Taq polymerases that decrease efficiency for barcoding, genomic library construction, or Southern blot analysis [26,27]. Although the CTAB-based DNA extraction method is widely used, it occasionally fails to remove all phenolics from DNA preparations, so antioxidants, such as β-mercaptoethanol and PVP, are commonly used to overcome problems related to phenolics [26].

Nevertheless, several modified CTAB methods [28] are available for DNA isolation of species-specific insects; however, in some instances, they have been erroneously referenced [29]. Moreover, the implementation of other published DNA extraction methods does not ensure high-quality DNA isolation from the different body structures of fall armyworm [30]; among these, CTAB total DNA extraction [31], [32], [33]. There is not even a consensus about the specific body section to be dissected to conduct a successful DNA extraction [28,[34], [35], [36]. In addition, several commercially available DNA extraction kits are recommended for insect DNA isolation, such as DNeasy Blood & Tissue Kit from Qiagen [37], DNAzol^Ⓡ^ Reagent [38], and Puregene^Ⓡ^ Kit [39]. These kits, however, are not suitable for DNA isolation of some insect species, particularly those with high polysaccharide, polyphenol, protein, and other cellular sub-products. More importantly they are costly per sample and produce low DNA yield in comparison with homemade protocols [40] such as SDS [34] or CTAB [26]. Previous research in the identification of *S. frugiperda* biotypes used a CTAB method to isolate DNA from the FAW and achieved success in only 10% of the analyzed samples [41] because of the low quality or degradation of the extracted samples. To improve quality, researchers used DNeasy Blood & Tissue Kit from Qiagen, mentioned a range of concentrations from 25 ng/µL to 100 ng/µL, and did not report *A_260_:A_280_* or *A_260_:A_230_* ratios.

Given the evident low success of the proposed CTAB method in other studies [26,^29^,^35^,40], the need to develop an optimized high-quality DNA isolation method for FAW, using all body sections and tissue from different storage conditions, is acknowledged. Thus, we developed a modified CTAB-based DNA isolation protocol that can produce high-yield/-quality total genomic DNA with minimal contamination using low-cost chemicals. Furthermore, our high-quality DNA isolation protocol is fast and simple and produces sufficient quantities of high-quality DNA.

## Materials and methods

### Biological materials

Different larval stages of *Spodoptera frugiperda* were sampled from four agricultural sub-regions of Colombia: the Magdalena River Valley, the Humid Caribbean, the Cauca River Valley, and the Orinoquía. A sub-sample of 64 was stored in 70% ethanol at 4 ᵒC. Additionally, adults from larvae collected in maize, which were stored dry for a year at 4 °C, were used for DNA extraction.

### Solutions, reagents, and supplies

All reagents and solutions were purchased at the required concentration from commercial suppliers, except for ribonuclease A from bovine pancreas (Merck; Cat. No. R4875) that was purchased as powder and prepared in a working solution of 10 mg/mL. The stock solutions of UltraPure™ 1 M Tris–HCl (pH 8.0; Thermo Fisher Scientific; Cat. No. 15568025), sodium chloride solution 5 M (Merck; Cat. No. S5150), UltraPure™ 0.5 M ethylenediamine tetraacetic acid (EDTA; pH 8.0; Promega; Cat. No. V4233), hexadecyltrimethylammonium bromide (CTAB; Merck; Cat. No. H5882), 2-mercaptoethanol (Sigma-Aldrich; Cat. No. M6250), UltraPure™ DNase/RNase-free distilled water (Thermo Fisher Scientific; Cat. No. 10977-015), chloroform-isoamyl-alcohol mixture 24:1 (Merck; Cat. No. C0549), absolute ethanol for analysis (Merck; Cat. No. 1.00983), chloroform for analysis (Merck; Cat. No. 366919), and 2-propanol (Merck; Cat. No. 109634) were of molecular biology grade and were free of RNAses, DNAses, and pyrogens. All disposable plasticware for the preparation of extraction buffer and the tubes used for extraction were free of RNAses, DNAses, and pyrogens.

### DNA extraction procedure

To obtain high-quality DNA from *S. frugiperda*, a total of four DNA isolation protocols were assessed (Table 1) [11,35]. The initial sample input for DNA extraction depended on the protocol: head, thorax, abdomen, and post-abdomen, in a weight range of 0.1 g to 1.0 g. Prior to DNA extraction, benchtop areas were cleaned using 70% ethanol and ELIMINase (Decon Laboratories Inc.; Cat. No. 1101). Pipettes were also cleaned using the same procedure and exposed to ultraviolet light for 30 min. Mortars and pestles were washed with soap, rinsed with 70% ethanol, and autoclaved at 118 ᵒC for 15 min.

**Table 1:** Summary of the main characteristics of the DNA extraction protocol implemented for fall armyworm in other studies.

Sample input	Protocol 1 [35,41]	Protocol 2 [29]	Protocol 3[36]	Protocol 4 [43]
	Head- abdominal section	Thorax	Abdomen	Post-abdomen
**Volume of buffer per sample (µL)**	400	400	500	400
**Extraction buffer**				

0.1 M Tris-HCL pH 8.0	**x**	**x**	**X**	**X**
1.4 M NaCl	**x**	**x**	**X**	**X**
0.02 M EDTA	**x**	**x**	**X**	**X**
2x CTAB	**x**	**x**	**X**	**X**
1% β-mercaptoethanol	**x**	**x**	**X**	**X**
PVP 1%				**X**
Proteinase K		**x**	**X**	**X**
**Incubation time (h)**	0.5	1.0	3.0	1.0
**Washing steps**				

Chloroform IAA x 2	**x**	**x**	**X**	**X**
Chloroform	**x**	**x**	**X**	**X**
Isopropanol	**x**	**x**	**X**	**X**
100% EtOH	**1**	**1**	**2**	**2**
70% EtOH	**1**			
**Purification step**				

10 mg/mL RNase A	X	x		x

All tissue samples (head, thorax, abdomen, and post-abdomen) were macerated and homogenized with extraction buffer pre-heated at 65 ᵒC. Proteinase K was added in the buffer according to other protocols (Table 1). Subsequently, all other steps were followed as described by each protocol (Table 1). Quantities and qualities of isolated DNA were evaluated spectrophotometrically by determining absorbance ratios of *A_260_:A_280_* and concentration using a Nanodrop^Ⓡ^ ND-1000 spectrophotometer (Thermo Fisher Scientific). DNA quality was further assessed electrophoretically on 1% agarose gels, which were prepared by adding 1.5 g of agar powder to 100 mL of 1X BS buffer (2% NaOH; 10% boric acid) and boiling until melted, followed by the addition of 4 µL of SYBR^Ⓡ^ Safe. Gels were run at 90 V for about 35 min.

## Results and discussion

**Fig. 1:**
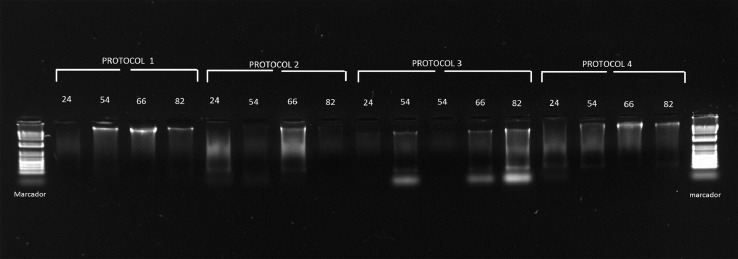
DNA-quality gel product of the implementation of the CTAB protocol for identification of *S. frugiperda* biotypes.

Initially, we extracted DNA from the head, thorax, abdomen, and post-abdomen separately according to previously described methods [29,^35^,^36^,42] (Table 1). However, the resulting DNA was of low yield and purity, and degraded in all cases when protocol 2 [28] or 3 [40] was used (Fig. 1, Table 2). These preliminary assays indicated that these protocols were not suitable for *Spodoptera* tissues, likely reflecting the prevalence of inhibitory compounds during the extraction process that are not efficiently removed during the washing steps proposed in the protocols and the lack of an efficient enzymatic lysis.

**Table 2:** Yields and *A_260_:A_280_* ratio of isolated total DNA from previously reported protocols and the protocol modified in this study.

Component	Protocol 1	Protocol 2	Protocol 3	Protocol 4	CIAT modified protocol
DNA yield rate (mean ± SE) (µg)	46 ± 15.15	63 ± 24.75	45 ± 25.66	47 ± 23.89	72 ± 16.66
Absorbance ratio (mean ± SE) and range	1.84 ± 0.08	1.45 ± 0.43	1.70 ± 0.12	1.77 ± 0.08	1.84 ± 0.07
Color of DNA pellet	light to dark brown	light to dark brown	light to dark brown	light brown to brown	light brown to brown
Estimated time (h)/sample	5.5	6.0	16.0	5.5	6.0

Therefore, according to the quality visualized in Fig. 1, to improve the quality of the DNA, we modified the methods reported [11,35] by adding mixing steps in a shaker to fully incorporate the organic compounds and increase the effectiveness of the washing steps, and also by increasing the precipitation time and improving the astringency of the RNase A treatment. The larval body section for DNA extraction must be dissected and liquid-nitrogen ground on the day of extraction, when samples have been stored with 70% ethanol. These stored FAW samples, once processed, cannot be placed back into ethanol and stored for later DNA extraction since they showed clear evidence of degradation. However, if the liquid-nitrogen ground samples are stored at −80 °C, they can be stored for several days before DNA extraction.

Our modified DNA isolation procedure was used for polymerase chain reaction (PCR), cloning, and sequencing to assess the quality and reliability of the DNA obtained. The protocol was used for all body segments, including samples collected from the field and reared in the laboratory. Here, we describe the modified protocol as follows:

Extraction buffer containing 100 mM Tris-HCL pH 8.0, 1.4 M NaCl, 0.02 M EDTA, 2x CTAB, and 1% 2-mercaptoethanol was prepared in ultra-pure water that was free of RNase and was then preheated to 65 °C prior to its use.

Tissues were (1) ground to a fine powder using a mortar and pestle in liquid nitrogen and stored at −80 °C until DNA isolation or (2) dissected and immediately hand-macerated using a pestle in a 1.5 mL tube with 200 µL of pre-heated extraction buffer for the homogenized sample. In this case, when the samples were macerated with liquid nitrogen, it was provided continuously to avoid melting of frozen tissues and to prevent DNA degradation until the addition of pre-heated buffer.

Pre-heated extraction buffer (400 µL) was added to fresh body part samples ground in liquid nitrogen; however, when the samples were either fresh tissue or tissue stored in 70% ethanol, they were macerated in 200 µL of pre-heated extraction buffer (in a 1.5 mL tube) to complete homogenization. The remaining 200 µL of pre-heated extraction buffer was added to the final extraction volume of 400 µL. After adding the extraction buffer, the samples were incubated for 30 min at 65 ᵒC and mixed by inversion every 5 min.

After incubation, one volume of chloroform-isoamyl-alcohol (24:1) was added to the samples and mixed by inversion at 90 rpm for 1 min in a shaker (VWR^Ⓡ^ Mini Shaker). Later, the samples were centrifuged at 18,700 rcf for 6 min at room temperature (Eppendorf Centrifuge 5427 R, Germany). Then, the supernatant was transferred to a new sterile 1.5 mL tube, to which one volume of chloroform was added and mixed thoroughly by inversion for 1 min, followed by a centrifugation at room temperature (18,700 rcf). This step was repeated one more time.

The resulting supernatant was then transferred to a new sterile 1.5 mL tube with care not to disturb the lower chloroform phases. Then, one volume of cold (−20 °C) 2-propanol was added. The samples were incubated for ≥2 h at −20 °C and, after that, centrifuged at 18,700 rcf for 12 min at 4 °C. After discarding the supernatants, precipitated pellets were washed immediately with 200 µL of cold (−20 °C) 100% ethanol and centrifuged at maximum speed for 6 min. The pellets were then washed with 200 µL of cold (−20 °C) 70% ethanol. Finally, the supernatants were discarded, the pellets dried at room temperature for 1 h, resuspended in 50 µL of T_10_E_1_ and 5 µL of RNase A (10 mg/mL), incubated for 1 h at 37 °C, and stored at −20 °C.

**Fig. 2:**
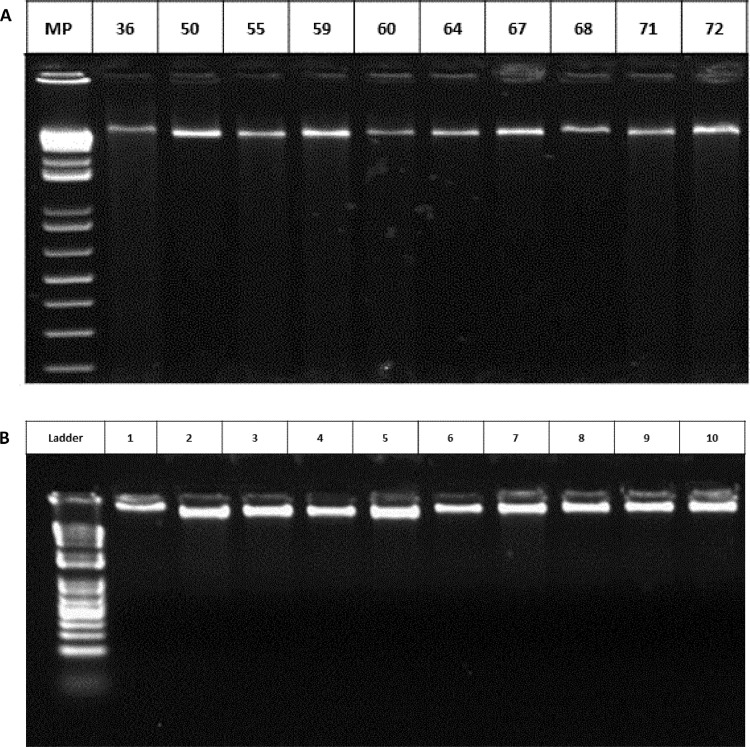
Quality gel of the DNA isolated using the modifications to the CTAB-based protocol proposed here. (A) DNA from FAW samples collected in field and stored in 70% ethanol. (B) DNA from hind legs of FAW adults subjected to long-time storage at 4 ᵒC.

The protocol described above was used on (1) the whole FAW larval body (Fig. 2.A, wells 36, 50, 55, 59, 60), (2) head tissue (Fig. 2.A, wells 64, 67, 68, 71, 72) stored with 70% ethanol, and (3) in posterior legs of FAW adults stored for 1 year at 4 °C (Fig. 2.B). Quantitative spectrometric assessments of DNA reached *A_260_:A_280_* ratios above 1.8, indicating very low protein contamination (Table 2). Qualitative assessment of the DNA in an agarose gel did not depict evidence of degradation (Fig. 2). In addition, the extraction protocol described here efficiently yielded 30–99 µg/g of high-quality total DNA from all samples, under several storage conditions. These yields were higher than with all other methods tested here (Table 2).

Although very few commercially available DNA isolation and purification methods reported acceptable concentrations and absorbance ratios, very few kits have been designed exclusively for use in insects [43]. Our modified CTAB method resulted in higher DNA yield (ng DNA vs. mg tissue) and quality when assessed on FAW body tissue. This protocol becomes a suitable option, especially when a large number of samples need to be extracted or if DNA of high concentration and quality is required for downstream molecular applications.

On the basis of our proposed protocol, EDTA starts cell wall rupture to release the nucleic acids [44]. Tris-HCl equilibrates the pH (close to 8.0), ensuring lysis. β-mercaptoethanol delays sample oxidation, facilitating DNA recovery. On the other hand, the addition of β-mercaptoethanol helps to reduce browning in DNA preparations produced by the oxidation of phenolics. Sodium chloride is efficient in extracting nucleic acids from polysaccharides [45,46]. Cetyl trimethylammonium bromide (CTAB) in buffer has been used to obtain good quality, given that it acts on the membrane lipid layer to remove proteins. Chloroform-isoamyl-alcohol washing promoted protein precipitation and decreased polysaccharide contents. We also performed two chloroform washes to remove debris and proteins in the initial stages of DNA isolation. The mixing step with chloroform-isoamyl-alcohol in the shaker enables the disruption of the cell wall, which improves enzymatic lysis, decreases the chances of DNA fragmentation, and ensures a high DNA yield. The longer incubation period with RNase A decreases the possibility to obtain absorbance ratios lower than 1.9, which can be caused by RNA contamination. These observations suggest that CTAB and its modifications increase DNA yields and eliminate possible contamination with lipids, proteins, and other cellular compounds that may interfere with the downstream DNA purposes.

**Fig. 3:**
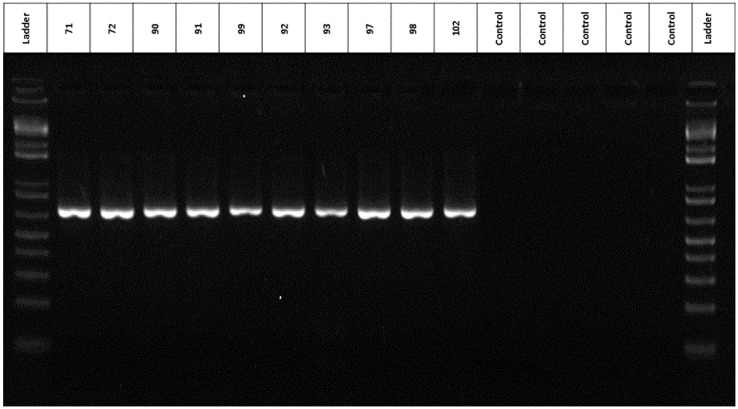
Amplification of the COI* region of FAW DNA isolated by the application of the DNA extraction protocol modified and proposed here. (*) Using as a template 10 ng/µL isolated with the modified protocol implemented in this study. The negative controls correspond to the control without template to rule out the presence of cross contamination.

**Fig. 4:**
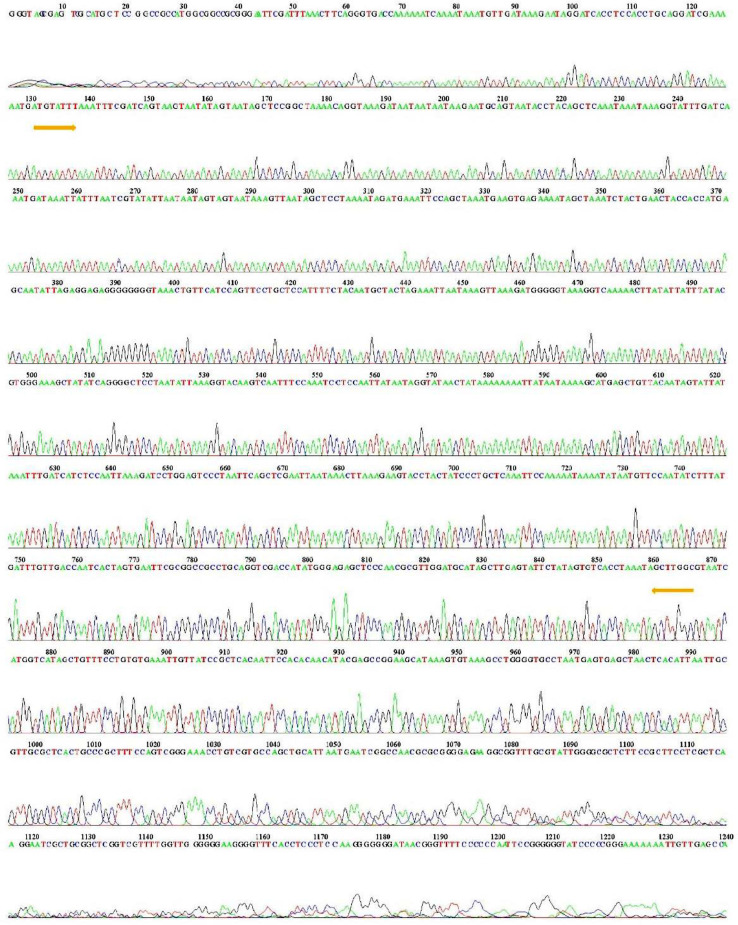
Resulting chromatogram of the COI region sequencing. The yellow arrows show the relative position of the primers flanking the COI fragment of interest.

Using the DNA obtained from our modified protocol, we amplified the cytochrome oxidase I region (COI) [45] to evaluate FAW biotype composition and diversity (Fig. 3) in 83 field populations. The COI gene is an ideal candidate to be used as a DNA barcode system. Its amplification and sequencing are useful when rapid detection and identification of a pest are required for regulatory purposes [47,48]. The targeted DNA fragment was cloned and sent to Macrogen (Korea) for sequencing. The resulting chromatogram depicted evenly-spaced peaks, each with only one color (Fig. 4). Little baseline noise, in a few cases, was present, but it was minimal. Even so, the real peaks were still easy to call. The fragments sequenced were blasted against the NCBI database and the sequences had a 99% similarity with previously reported sequences of *S. frugiperda* in other experiments [49]. There was no contamination of the sequences that allowed associating the sequences with other species.

In conclusion, we optimized a protocol to isolate high-quality DNA from FAW tissues, achieving high yields and DNA integrity. The extractions carried out confirmed that the implementation of this protocol for the isolation of DNA allows the selection of DNA samples free of inhibitors and generating a suitable product for PCR amplification, for its subsequent cloning and sequencing. It was determined that this protocol is successful in all body sections of FAW larvae when the specimens are collected in 70% ethanol and in adult legs when they are stored at 4 °C. A total duration of 6 h was established for the implementation of this protocol, of which 3 h correspond to the incubation period. The total number of samples that can be processed per day varies between 24 when the tissue is dissected within the same day just before DNA isolation from samples stored in 70% and 96% ethanol, and 96 when the tissue was previously ground in liquid nitrogen and stored at −80 °C until DNA isolation. Finally, the quality of the DNA obtained suggests that this protocol can be implemented as a tool for the detection of *Spodoptera* species and biotypes.

## Declaration of Competing Interest

The Authors confirm that there are no conflicts of interest.

## Appendix Supplementary materials

Image, application 1
