# Supplementary material for: An optimized high-quality DNA isolation protocol for spodoptera frugiperda J. E. smith (Lepidoptera: Noctuidae)
Source: MethodsX. 2021 Feb 3;8:101255. doi: 10.1016/j.mex.2021.101255 (PMC8374285; doi:10.1016/j.mex.2021.101255)
Supplement: Supplementary file 1 [file mmc1.docx]

**Supplementary material *and/or* Additional information:** *[OPTIONAL. We also give you the option to submit both supplementary material and additional information. Supplementary material relates directly to the work that you have submitted and can include extensive excel tables, raw data etc. We would also encourage you to include failed methods or describe adjustments to your methods that did not work. Additional information can include anything else that is not directly related to your method, e.g. more general background information, useful links etc. Introduction is not a section included in the MethodsX format. This information could be moved to the end under Additional Information.*
